# On Satisficing in Quantitative Games

**DOI:** 10.1007/978-3-030-72016-2_2

**Published:** 2021-03-01

**Authors:** Suguman Bansal, Krishnendu Chatterjee, Moshe Y. Vardi

**Affiliations:** 8 Eindhoven University of Technology, Eindhoven, The Netherlands; 9 Aalborg University, Aalborg East, Denmark; 10 University of Pennsylvania, Philadelphia, USA; 11 IST Austria, Klosterneuburg, Austria; 12 Rice University, Houston, USA

## Abstract

Several problems in planning and reactive synthesis can be reduced to the analysis of two-player quantitative graph games. *Optimization* is one form of analysis. We argue that in many cases it may be better to replace the optimization problem with the *satisficing problem*, where instead of searching for optimal solutions, the goal is to search for solutions that adhere to a given threshold bound.

This work defines and investigates the satisficing problem on a two-player graph game with the discounted-sum cost model. We show that while the satisficing problem can be solved using numerical methods just like the optimization problem, this approach does not render compelling benefits over optimization. When the discount factor is, however, an integer, we present another approach to satisficing, which is purely based on automata methods. We show that this approach is algorithmically more performant – both theoretically and empirically – and demonstrates the broader applicability of satisficing over optimization.

## 1 Introduction

*Quantitative properties* of systems are increasingly being explored in automated reasoning [4,14,16,20,21,26]. In decision-making domains such as planning and reactive synthesis, quantitative properties have been deployed to describe *soft constraints* such as quality measures [11], cost and resources [18,22], rewards [31], and the like. Since these constraints are soft, it suffices to generate solutions that are *good enough* w.r.t. the quantitative property.

Existing approaches on the analysis of quantitative properties have, however, primarily focused on *optimization* of these constraints, i.e., to generate optimal solutions. We argue that there may be disadvantages to searching for optimal solutions, where *good enough* ones may suffice. First, optimization may be more expensive than searching for good-enough solutions. Second, optimization restricts the search-space of possible solutions, and thus could limit the broader applicability of the resulting solutions. For instance, to generate solutions that operate *within* battery life, it is too restrictive to search for solutions with *minimal* battery consumption. Besides, solutions with minimal battery consumption may be limited in their applicability, since they may not satisfy other goals, such as desirable temporal tasks.

To this end, this work focuses on directly searching for good-enough solutions. We propose an alternate form of analysis of quantitative properties in which the objective is to search for a solution that adheres to *a given threshold bound*, possibly derived from a physical constraint such as battery life. We call this the *satisficing problem*, a term popularized by H.A.Simon in economics to mean *satisfy and suffice*, implying a search for good-enough solutions [1]. Through theoretical and empirical investigation, we make the case that satisficing is algorithmically more performant than optimization and, further, that satisficing solutions may have broader applicability than optimal solutions.

This work formulates and investigates the satisficing problem on two-player, finite-state games with the discounted-sum (DS) cost model, which is a standard cost-model in decision-making domains [24,25,28]. In these games, players take turns to pass a token along the *transition relation* between the states. As the token is pushed around, the play accumulates costs along the transitions using the DS cost model. The players are assumed to have opposing objectives: one player maximizes the cost, while the other player minimizes it. We define the satisficing problem as follows: *Given a* threshold value $v \in \mathbb{Q}$*, does there exist a strategy for the minimizing (or maximizing) player that ensures the cost of all resulting plays is strictly or non-strictly lower (or greater) than the threshold* $v$*?*

Clearly, the satisficing problem is decidable since the optimization problem on these quantitative games is known to be solvable in pseudo-polynomial time [17,23,32]. To design an algorithm for satisficing, we first adapt the celebrated value-iteration (VI) based algorithm for optimization [32] (§ 3). We show, however, that this algorithm, called VISatisfice, displays the same complexity as optimization and hence renders no complexity-theoretic advantage. To obtain worst-case complexity, we perform a thorough worst-case analysis of VI for optimization. It is interesting that a thorough analysis of VI for optimization had hitherto been absent from the literature, despite the popularity of VI. To address this gap, we first prove that VI should be executed for $\Theta(|V|)$ iterations to compute the optimal value, where $V$ and $E$ refer to the sets of states and transitions in the quantitative game. Next, to compute the overall complexity, we take into account the cost of arithmetic operations as well, since they appear in abundance in VI. We demonstrate an orders-of-magnitude difference between the complexity of VI under different cost-models of arithmetic. For instance, for integer discount factors, we show that VI is $\mathcal{O}(|V| \cdot |E|)$ and $\mathcal{O}(|V|^2 \cdot |E|)$ under the unit-cost and bit-cost models of arithmetic, respectively. Clearly, this shows that VI for optimization, and hence VISatisfice, does not scale to large quantitative games.

We then present a purely automata-based approach for satisficing (§ 4). While this approach applies to integer discount factors only, it solves satisficing in $\mathcal{O}(|V| + |E|)$ time. This shows that there is a fundamental separation in complexity between satisficing and VI-based optimization, as even the lower bound on the number of iterations in VI is higher. In this approach, the satisficing problem is reduced to solving a safety or reachability game. Our core observation is that the criteria to fulfil satisficing with respect to threshold value $v \in \mathbb{Q}$ can be expressed as membership in an automaton that accepts a weight sequence $A$ iff $DS(A, d)\ \mathsf{R}\ v$ holds, where $d > 1$ is the discount factor and $\mathsf{R} \in \{\leq, \geq, <, >\}$. In existing literature, such automata are called *comparator automata* (comparators, in short) when the threshold value $v = 0$ [6,7]. They are known to have a compact safety or co-safety automaton representation [9,19], which could be used to reduce the satisficing problem with zero threshold value. To solve satisficing for arbitrary threshold values $v \in Q$, we extend existing results on comparators to permit arbitrary but fixed threshold values $v \in \mathbb{Q}$. An empirical comparison between the performance of VISatisfice, VI for optimization, and automata-based solution for satisficing shows that the latter outperforms the others in efficiency, scalability, and robustness.

In addition to improved algorithmic performance, we demonstrate that satisficing solutions have broader applicability than optimal ones (§ 5). We examine this with respect to their ability to extend to temporal goals. That is, the problem is to find optimal/satisficing solutions that also satisfy a given temporal goal. Prior results have shown this to not be possible with optimal solutions [13]. In contrast, we show satisficing extends to temporal goals when the discount factor is an integer. This occurs because both satisficing and satisfaction of temporal goals are solved via automata-based techniques, which can be easily integrated.

In summary, this work contributes to showing that satisficing has algorithmic and applicability advantages over optimization in (deterministic) quantitative games. In particular, we have shown that the automata-based approach for satisficing have advantages over approaches in numerical methods like value-iteration. This gives yet another evidence in favor of automata-based quantitative reasoning and opens up several compelling directions for future work.

## 2 Preliminaries

### 2.1 Two-player graph games

*Reachability and safety games.* Both *reachability* and *safety games* are defined over the structure $G = (V = V_0 \uplus V_1, v_{\mathsf{init}}, E, \mathcal{F})$ [30]. It consists of a directed graph $(V, E)$, and a partition $(V_0, V_1)$ of its states $V$. State $v_{\mathsf{init}}$ is the *initial state* of the game. The set of successors of state $v$ is designated by $vE$. For convenience, we assume that every state has at least one outgoing edge, i.e, $vE \neq \emptyset$ for all $v \in V$. $\mathcal{F} \subseteq V$ is a non-empty set of states. $\mathcal{F}$ is referred to as *accepting* and *rejecting* states in reachability and safety games, respectively.

A *play* of a game involves two players, denoted by $P_0$ and $P_1$, to create an infinite path by moving a token along the transitions as follows: At the beginning, the token is at the initial state. If the current position $v$ belongs to $V_i$, then $P_i$ chooses the successor state from $vE$. Formally, a play $\rho = v_0 v_1 v_2 \dots$ is an infinite sequence of states such that the first state $v_0 = v_{\mathsf{init}}$, and each pair of successive states is a transition, i.e., $(v_k, v_{k+1}) \in E$ for all $k \geq 0$. A play is *winning for player* $P_1$ in a reachability game if it visits an accepting state, and *winning for player* $P_0$ otherwise. The opposite holds in safety games, i.e., a play is winning for player $P_1$ if it does not visit any rejecting state, and winning for $P_0$ otherwise.

A *strategy* for a player is a recipe that guides the player on which state to go next to based on the history of the play. A *strategy is winning for a player* $P_i$ if for all strategies of the opponent player $P_{1-i}$, the resulting plays are winning for $P_i$. To *solve* a graph game means to determine whether there exists a winning strategy for player $P_1$. Reachability and safety games are solved in $\mathcal{O}(|V|+|E|)$.

*Quantitative graph games.* A *quantitative graph game* (or quantitative game, in short) is defined over a structure $G = (V = V_0 \uplus V_1, v_{\mathsf{init}}, E, \gamma)$. $V$, $V_0$, $V_1$, $v_{\mathsf{init}}$, $E$, plays and strategies are defined as earlier. Each transition of the game is associated with a *cost* determined by the *cost function* $\gamma : E \to \mathbb{Z}$. The *cost sequence* of a play $\rho$ is the sequence of costs $w_0 w_1 w_2 \dots$ such that $w_k = \gamma((v_k, v_{k+1}))$ for all $i \geq 0$. Given a discount factor $d > 1$, the *cost of play* $\rho$, denoted $wt(\rho)$, is the discounted sum of its cost sequence, i.e., $wt(\rho) = DS(\rho, d) = w_0 + \frac{w_1}{d} + \frac{w_2}{d^2} + \dots$.

### 2.2 Automata and formal languages

*Büchi automata.* A *Büchi automaton* is a tuple $\mathcal{A} = (S,\ \Sigma,\ \delta,\ s_{\mathcal{I}},\ \mathcal{F})$, where $S$ is a finite set of *states*, $\Sigma$ is a finite *input alphabet*, $\delta \subseteq (S \times \Sigma \times S)$ is the *transition relation*, state $s_{\mathcal{I}} \in S$ is the *initial state*, and $\mathcal{F} \subseteq S$ is the set of *accepting states* [30]. A Büchi automaton is *deterministic* if for all states $s$ and inputs $a$, $|\{s'|(s, a, s') \in \delta \text{ for some } s'\}| \leq 1$. For a word $w = w_0 w_1 \dots \in \Sigma^\omega$, a *run* $\rho$ of $w$ is a sequence of states $s_0 s_1 \dots$ s.t. $s_0 = s_{\mathcal{I}}$, and $\tau_i = (s_i, w_i, s_{i+1}) \in \delta$ for all $i$. Let $\mathit{inf}(\rho)$ denote the set of states that occur infinitely often in run $\rho$. A run $\rho$ is an *accepting run* if $\mathit{inf}(\rho) \cap \mathcal{F} \neq \emptyset$. A word $w$ is an accepting word if it has an accepting run. The language of Büchi automaton $\mathcal{A}$ is the set of all words accepted by $\mathcal{A}$. Languages accepted by Büchi automata are called *$\omega$-regular.*

*Safety and co-safety languages.* Let $\mathcal{L} \subseteq \Sigma^\omega$ be a language over alphabet $\Sigma$. A finite word $w \in \Sigma^*$ is a *bad prefix* for $\mathcal{L}$ if for all infinite words $y \in \Sigma^\omega$, $x \cdot y \notin \mathcal{L}$. A language $\mathcal{L}$ is a *safety language* if every word $w \notin \mathcal{L}$ has a bad prefix for $\mathcal{L}$ [3]. A *co-safety language* is the complement of a safety language [19]. Safety and co-safety languages that are $\omega$-regular are represented by specialized Büchi automata called *safety* and *co-safety automata*, respectively.

*Comparison language and comparator automata.* Given integer bound $\mu > 0$, discount factor $d > 1$, and relation $\mathsf{R} \in \{<, >, \leq, \geq, =, \neq\}$ the *comparison language with upper bound $\mu$, relation* $\mathsf{R}$*, discount factor $d$* is the language of words over the alphabet $\Sigma = \{-\mu, \dots, \mu\}$ that accepts $A \in \Sigma^\omega$ iff $DS(A, d)\ \mathsf{R}\ 0$ holds [5,9]. The *comparator automata with upper bound $\mu$, relation* $\mathsf{R}$*, discount factor $d$* is the automaton that accepts the corresponding comparison language [6]. Depending on $\mathsf{R}$, these languages are safety or co-safety [9]. A comparison language is said to be *$\omega$-regular* if its automaton is a Büchi automaton. Comparison languages are $\omega$-regular iff the discount factor is an integer [7].

## 3 Satisficing via Optimization

This section shows that there are no complexity-theoretic benefits to solving the satisficing problem via algorithms for the optimization problem.

§ 3.1 formally defines the satisficing problem and reviews the celebrated value-iteration (VI) algorithm for optimization by Zwick and Patterson (ZP). While ZP *claim without proof* that the algorithm runs in pseudo-polynomial time [32], its worst-case analysis is absent from literature. This section presents a detailed account of the said analysis, and exposes the dependence of VI's worst-case complexity on the discount factor $d > 1$ and the cost-model for arithmetic operations i.e. unit-cost or bit-cost model. The analysis is split into two parts: First, § 3.2 shows it is sufficient to terminate after a finite-number of iterations. Next, § 3.3 accounts for the cost of arithmetic operations per iteration to compute VI's worst-case complexity under unit- and bit-cost cost models of arithmetic Finally, § 3.4 presents and analyzes our VI-based algorithm for satisficing VISatisfice.

### 3.1 Satisficing and Optimization

**Definition 1 (Satisficing problem).** *Given a quantitative graph game $G$ and a threshold value $v \in \mathbb{Q}$, the* satisficing problem *is to determine whether the minimizing (or maximizing) player has a strategy that ensures the cost of all resulting plays is strictly or non-strictly lower (or greater) than the threshold $v$.*

The satisficing problem can clealy be solved by solving the *optimization problem*. The optimal cost of a quantitative game is that value such that the maximizing and minimizing players can guarantee that the cost of plays is at least and at most the optimal value, respectively.

**Definition 2 (Optimization problem).** *Given a quantitative graph game $G$, the* optimization problem *is to compute the optimal cost from all possible plays from the game, under the assumption that the players have opposing objectives to maximize and minimize the cost of plays, respectively.*

Seminal work by Zwick and Patterson showed the optimization problem is solved by the value-iteration algorithm presented here [32]. Essentially, the algorithm plays a min-max game between the two players. Let $wt_k(v)$ denote the optimal cost of a $k$-length game that begins in state $v \in V$. Then $wt_k(v)$ can be computed using the following equations: The optimal cost of a 1-length game beginning in state $v \in V$ is $\max\{\gamma(v, w) | (v, w) \in E\}$ if $v \in V_0$ and $\min\{\gamma(v, w) | (v, w) \in E\}$ if $v \in V_1$. Given the optimal-cost of a $k$-length game, the optimal cost of a $(k+1)$-length game is computed as follows:

$$wt_{k+1}(v) = \begin{cases} max\{\gamma(v, w) + \frac{1}{d} \cdot wt_k(w) | (v, w) \in E\} \text{ if } v \in V_0 \\ min\{\gamma(v, w) + \frac{1}{d} \cdot wt_k(w) | (v, w) \in E\} \text{ if } v \in V_1 \end{cases}$$

Let $W$ be the optimal cost. Then, $W = \lim_{k \to \infty} wt_k(v_{\mathsf{init}})$. [27,32].

### 3.2 VI: Number of iterations

The VI algorithm described above terminates at *infinitum.* To compute the algorithms' worst-case complexity, we establish a linear bound on the number of iterations that is sufficient to compute the optimal cost. We also establish a matching lower bound, showing that our analysis is tight.

*Upper bound on number of iterations.* The upper bound computation utilizes one key result from existing literature: There exist memoryless strategies for both players such that the cost of the resulting play is the optimal cost [27]. Then, there must exists an optimal play in the form of a *simple lasso* in the quantitative game, where a *lasso* is a play represented as $v_0v_1 \dots v_n(s_0s_2 \dots s_m)^\omega$. We call the initial segment $v_0v_1 \dots v_n$ its *head*, and the cycle segment $s_0s_1 \dots s_m$ its *loop*. A lasso is *simple* if each state in $\{v_0 \dots v_n, s_0, \dots s_m\}$ is distinct. We begin our proof by assigning constraints on the optimal cost using the simple lasso structure of an optimal play (Corollary 1 and Corollary 2).

Let $l = a_0 \dots a_n(b_0 \dots b_m)^\omega$ be the cost sequence of a lasso such that $l_1 = a_0 \dots a_n$ and $l_2 = b_0 \dots b_m$ are the cost sequences of the head and the loop, respectively. Then the following can be said about $DS(l_1 \cdot l_2^\omega, d)$,

**Lemma 1.** *Let $l = l_1 \cdot (l_2)^\omega$ represent an integer cost sequence of a lasso, where $l_1$ and $l_2$ are the cost sequences of the head and loop of the lasso. Let $d = \frac{p}{q}$ be the discount factor. Then, $DS(l, d)$ is a rational number with denominator at most $(p^{|l_2|} - q^{|l_2|}) \cdot (p^{|l_1|})$.*

Lemma 1 is proven by unrolling $DS(l_1 \cdot l_2^\omega, d)$. Then, the first constraint on the optimal cost is as follows:

**Corollary 1.** *Let $G = (V, v_{\mathsf{init}}, E, \gamma)$ be a quantitative graph game. Let $d = \frac{p}{q}$ be the discount factor. Then the optimal cost of the game is a rational number with denominator at most $(p^{|V|} - q^{|V|}) \cdot (p^{|V|})$*

*Proof.* Recall, there exists a simple lasso that computes the optimal cost. Since a simple lasso is of $|V|$-length at most, the length of its head and loop are at most $|V|$ each. So, the expression from Lemma 1 simplifies to $(p^{|V|} - q^{|V|}) \cdot (p^{|V|})$. □

The second constraint has to do with the minimum non-zero difference between the cost of simple lassos:

**Corollary 2.** *Let $G = (V, v_{\mathsf{init}}, E, \gamma)$ be a quantitative graph game. Let $d = \frac{p}{q}$ be the discount factor. Then the minimal non-zero difference between the cost of simple lassos is a rational with denominator at most $(p^{(|V|)} - q^{(|V|)})^2 \cdot (p^{(2 \cdot |V|)})$.*

*Proof.* Given two rational numbers with denominator at most $a$, an upper bound on the denominator of minimal non-zero difference of these two rational numbers is $a^2$. Then, using the result from Corollary 1, we immediately obtain that the minimal non-zero difference between the cost of two lassos is a rational number with denominator at most $(p^{(|V|)} - q^{(|V|)})^2 \cdot (p^{(2 \cdot |V|)})$. □

For notational convenience, let $\mathsf{bound}_W = (p^{|V|} - q^{|V|}) \cdot (p^{|V|})$ and $\mathsf{bound}_{\mathsf{diff}} = (p^{(|V|)} - q^{(|V|)})^2 \cdot (p^{(2 \cdot |V|)})$. Wlog, $|V| > 1$. Since, $\frac{1}{\mathsf{bound}_{\mathsf{diff}}} < \frac{1}{\mathsf{bound}_W}$, there is at most one rational number with denominator $\mathsf{bound}_\mathsf{W}$ or less in any interval of size $\frac{1}{\mathsf{bound}_{\mathsf{diff}}}$. Thus, if we can identify an interval of size less than $\frac{1}{\mathsf{bound}_{\mathsf{diff}}}$ around the optimal cost, then due to Corollary 1, the optimal cost will be the unique rational number with denominator $\mathsf{bound}_\mathsf{W}$ or less in this interval.

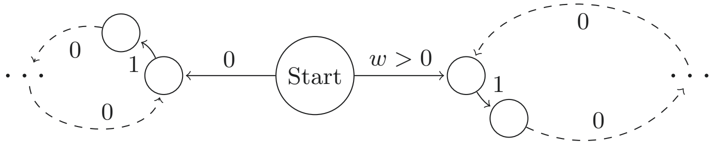

**Fig. 1.** Sketch of game graph which requires $\Omega(|V|)$ iterations

Thus, the final question is to identify a small enough interval (of size $\frac{1}{\mathsf{bound}_{\mathsf{diff}}}$ or less) such that the optimal cost lies within it. To find an interval around the optimal cost, we use a finite-horizon approximation of the optimal cost:

**Lemma 2.** *Let $W$ be the optimal cost in quantitative game $G$. Let $\mu > 0$ be the maximum of absolute value of cost on transitions in $G$. Then, for all $k \in \mathbb{N}$,*

$$wt_k(v_{\mathsf{init}}) - \frac{1}{d^{k-1}} \cdot \frac{\mu}{d-1} \leq W \leq wt_k(v_{\mathsf{init}}) + \frac{1}{d^{k-1}} \cdot \frac{\mu}{d-1}$$

*Proof.* Since $W$ is the limit of $wt_k(v_{\mathsf{init}})$ as $k \to \infty$, $W$ must lie in between the minimum and maximum cost possible if the $k$-length game is extended to an infinite-length game. The minimum possible extension would be when the $k$-length game is extended by iterations in which the cost incurred in each round is $-\mu$. Therefore, the minimum possible value is $wt_k(v_{\mathsf{init}}) - \frac{1}{d^{k-1}} \cdot \frac{\mu}{d-1}$. Similarly, the maximum possible value is $wt_k(v_{\mathsf{init}}) + \frac{1}{d^{k-1}} \cdot \frac{\mu}{d-1}$. □

Now that we have an interval around the optimal cost, we can compute the number of iterations of VI required to make it smaller than $1/\mathsf{bound}_{\mathsf{diff}}$.

**Theorem 1.** *Let $G = (V, v_{\mathsf{init}}, E, \gamma)$ be a quantitative graph game. Let $\mu > 0$ be the maximum of absolute value of costs along transitions. The number of iterations required by the value-iteration algorithm is*

1. *$\mathcal{O}(|V|)$ when discount factor $d \geq 2$,*
2. *$\mathcal{O}\Big(\frac{\log(\mu)}{d-1} + |V|\Big)$ when discount factor $1 < d < 2$.*

*Proof (Sketch).* As discussed in Corollary 1-2 and Lemma 2, the optimal cost is the unique rational number with denominator $\frac{1}{\mathsf{bound}_W}$ or less within the interval $(wt_k(v_{\mathsf{init}}) - \frac{1}{d^{k-1}} \cdot \frac{\mu}{d-1}, wt_k(v_{\mathsf{init}}) + \frac{1}{d^{k-1}} \cdot \frac{\mu}{d-1})$ for a large enough $k > 0$ such that the interval's size is less than $\frac{1}{\mathsf{bound}_{\mathsf{diff}}}$. Thus, our task is to determine the value of $k > 0$ such that $2 \cdot \frac{\mu}{d-1 \cdot d^{k-1}} \leq \frac{1}{\mathsf{bound}_{\mathsf{diff}}}$ holds. The case $d \geq 2$ is easy to simplify. The case $1 < d < 2$ involves approximations of logarithms of small values. □

*Lower bound on number of iterations of VI.* We establish a matching lower bound of $\Omega(|V|)$ iterations to show that our analysis is tight.

Consider the sketch of a quantitative game in Fig 1. Let all states belong to the maximizing player. Hence, the optimization problem reduces to searching for a *path* with optimal cost. Now let the loop on the right-hand side (RHS) be larger than the loop on the left-hand side (LHS). For carefully chosen values of

$w$ and lengths of the loops, one can show that the path for optimal cost of a $k$-length game is along the RHS loop when $k$ is small, but along the LHS loop when $k$ is large. This way, the correct maximal value can be obtained only at a large value for $k$. Hence the VI algorithm runs for at least enough iterations that the optimal path will be in the LHS loop. By meticulous reverse engineering of the size of both loops and the value of $w$, one can guarantee that $k = \Omega(|V|)$.

### 3.3 Worst-case complexity analysis of VI for optimization

Finally, we complete the worst-case complexity analysis of VI for optimization. We account for the the cost of arithmetic operations since they appear in abundance in VI. We demonstrate that there are orders-of-magnitude of difference in complexity under different models of arithmetic, namely unit-cost and bit-cost.

*Unit-cost model.* Under the unit-cost model of arithmetic, all arithmetic operations are assumed to take constant time.

**Theorem 2.** *Let $G = (V, v_{\mathsf{init}}, E, \gamma)$ be a quantitative graph game. Let $\mu > 0$ be the maximum of absolute value of costs along transitions. The worst-case complexity of the optimization problem under unit-cost model of arithmetic is*

1. $\mathcal{O}(|V| \cdot |E|)$ *when discount factor* $d \geq 2$,
2. $\mathcal{O}\Big(\frac{\log(\mu)\cdot|E|}{d-1} + |V| \cdot |E|\Big)$ *when discount factor* $1 < d < 2$.

*Proof.* Each iteration takes $\mathcal{O}(E)$ cost since every transition is visited once. Thus, the complexity is $\mathcal{O}(|E|)$ multiplied by the number of iterations (Theorem 1). □

*Bit-cost model.* Under the bit-cost model, the cost of arithmetic operations depends on the size of the numerical values. Integers are represented in their bit-wise representation. Rational numbers $\frac{r}{s}$ are represented as a tuple of the bit-wise representation of integers $r$ and $s$. For two integers of length $n$ and $m$, the cost of their addition and multiplication is $O(m + n)$ and $O(m \cdot n)$, respectively.

**Theorem 3.** *Let $G = (V, v_{\mathsf{init}}, E, \gamma)$ be a quantitative graph game. Let $\mu > 0$ be the maximum of absolute value of costs along transitions. Let $d = \frac{p}{q} > 1$ be the discount factor. The worst-case complexity of the optimization problem under the bit-cost model of arithmetic is*

1. $\mathcal{O}(|V|^2 \cdot |E| \cdot \log p \cdot \max\{\log \mu, \log p\})$ *when* $d \geq 2$,
2. $\mathcal{O}\Big(\Big(\frac{\log(\mu)}{d-1} + |V|\Big)^2 \cdot |E| \cdot \log p \cdot \max\{\log \mu, \log p\}\Big)$ *when* $1 < d < 2$.

*Proof (Sketch).* Since arithmetic operations incur a cost and the length of representation of intermediate costs increases linearly in each iteration, we can show that the cost of conducting the $j$-th iteration is $\mathcal{O}(|E| \cdot j \cdot \log \mu \cdot \log p)$. Their summation will return the given expressions. □

*Remarks on integer discount factor.* Our analysis shows that when the discount factor is an integer ($d \geq 2$), VI requires $\Theta(|V|)$ iterations. Its worst-case complexity is, therefore, $\mathcal{O}(|V| \cdot |E|)$ and $\mathcal{O}(|V|^2 \cdot |E|)$ under the unit-cost and bit-cost models for arithmetic, respectively. From a practical point of view, the bit-cost model is more relevant since implementations of VI will use multi-precision libraries to avoid floating-point errors. While one may argue that the upper bounds in Theorem 3 could be tightened, they would not improve significantly due to the $\Omega(|V|)$ lower bound on number of iterations.

### 3.4 Satisficing via value-iteration

We present our first algorithm for the satisficing problem. It is an adaptation of VI. However, we see that it does not fare better than VI for optimization.

VI-based algorithm for satisficing is described as follows: Perform VI for optimization. Terminate as soon as one of these occurs: (a). VI completes as many iterations from Theorem 1, or (b). The threshold value falls outside the interval defined in Lemma 2. Either way, one can tell how the threshold value relates to the optimal cost to solve satisficing. Clearly, (a) needs as many iterations as optimization; (b) does not reduce the number of iterations since it is inversely proportional to the distance between optimal cost and threshold value:

**Theorem 4.** *Let $G = (V, v_{\mathsf{init}}, E, \gamma)$ be a quantitative graph game with optimal cost $W$. Let $v \in \mathbb{Q}$ be the threshold value. Then number of iterations taken by a VI-based algorithm for the satisficing problem is* $\min\{O(|V|), \log \frac{\mu}{|W|-v}\}$ *if* $d \geq 2$ *and* $\min\{\mathcal{O}\Big(\frac{\log(\mu)}{d-1} + |V|\Big), \log \frac{\mu}{|W|-v}\}$ *if* $1 < d < 2$.

Observe that this bound is tight since the lower bounds from optimization apply here as well. The worst-case complexity can be completed using similar computations from § 3.3. Since, the number of iterations is identical to Theorem 1, the worst-case complexity will be identical to Theorem 2 and Theorem 3, showing no theoretical improvement. However, its implementations may terminate soon for threshold values far from the optimal but it will retain worst-case behavior for ones closer to the optimal. The catch is since the optimal cost is unknown apriori, this leads to a highly variable and *non-robust* performance.

## 4 Satisficing via Comparators

Our second algorithm for satisficing is purely based on automata-methods. While this approach operates with integer discount factors only, it runs linearly in the size of the quantitative game. This is lower than the number of iterations required by VI, let alone the worst-case complexities of VI. This approach reduces satisficing to solving a safety or reachability game using comparator automata.

The intuition is as follows: Given threshold value $v \in \mathbb{Q}$ and relation $\mathsf{R}$, let the satisficing problem be to ensure cost of plays relates to $v$ by $\mathsf{R}$. Then, a play $\rho$ is *winning for satisficing with $v$ and* $\mathsf{R}$ if its cost sequence $A$ satisfies $DS(A, d)$ $\mathsf{R}$

$v$, where $d > 1$ is the discount factor. When $d$ is an integer and $v = 0$, this simply checks if $A$ is in the safety/co-safety comparator, hence yielding the reduction.

The caveat is the above applies to $v = 0$ only. To overcome this, we extend the theory of comparators to permit arbitrary threshold values $v \in \mathbb{Q}$. We find that results from $v = 0$ transcend to $v \in \mathbb{Q}$, and offer compact comparator constructions (§ 4.1). These new comparators are then used to reduce satisficing to develop an efficient and scalable algorithm (§ 4.2). Finally, to procure a well-rounded view of its performance, we conduct an empirical evaluation where we see this comparator-based approach outperform the VI approaches § 4.3.

### 4.1 Foundations of comparator automata with threshold $v \in \mathbb{Q}$

This section extends the existing literature on comparators with threshold value $v = 0$ [6,5,9] to permit non-zero thresholds. The properties we investigate are of safety/co-safety and $\omega$-regularity. We begin with formal definitions:

**Definition 3 (Comparison language with threshold $v \in \mathbb{Q}$).** *For an integer upper bound $\mu > 0$, discount factor $d > 1$, equality or inequality relation $\mathsf{R} \in \{<, >, \leq, \geq, =, \neq\}$, and a threshold value $v \in \mathbb{Q}$ the* comparison language with upper bound $\mu$, relation $\mathsf{R}$, discount factor $d$ and threshold value $v$ *is a language of infinite words over the alphabet $\Sigma = \{-\mu, \dots, \mu\}$ that accepts $A \in \Sigma^\omega$ iff $DS(A, d)$ $\mathsf{R}$ $v$ holds.*

**Definition 4 (Comparator automata with threshold $v \in \mathbb{Q}$).** *For an integer upper bound $\mu > 0$, discount factor $d > 1$, equality or inequality relation $\mathsf{R} \in \{<, >, \leq, \geq, =, \neq\}$, and a threshold value $v \in \mathbb{Q}$ the* comparator automata with upper bound $\mu$, relation $\mathsf{R}$, discount factor $d$ and threshold value $v$ *is an automaton that accepts the DS comparison language with upper bound $\mu$, relation $\mathsf{R}$, discount factor $d$ and threshold value $v$.*

**Safety and co-safety of comparison languages.** The primary observation is that to determine if $DS(A, d)$ $\mathsf{R}$ $v$ holds, it should be sufficient to examine finite-length prefixes of $A$ since weights later on get heavily discounted. Thus,

**Theorem 5.** *Let $\mu > 1$ be the integer upper bound. For arbitrary discount factor $d > 1$ and threshold value $v \in \mathbb{Q}$*

1. *Comparison languages are safety languages for relations $R \in \{\leq, \geq, =\}$.*
2. *Comparison language are co-safety languages for relations $R \in \{<, >, \neq\}$.*

*Proof.* The proof is identical to that for threshold value $v = 0$ from [9]. □

**Regularity of comparison languages.** Prior work on threshold value $v = 0$ shows that a comparator is $\omega$-regular iff the discount factor is an integer [7]. We show the same result for arbitrary threshold values $v \in \mathbb{Q}$.

First of all, trivially, comparators with arbitrary threshold value are not $\omega$-regular for non-integer discount factors, since that already holds when $v = 0$.

The rest of this section proves $\omega$-regularity with arbitrary threshold values for integer discount factors. But first, let us introduce some notations: Since $v \in \mathbb{Q}$, w.l.o.g. we assume that the it has an $n$-length representation $v = v[0]v[1]\dots v[m](v[m+1]v[m+2]\dots v[n])^\omega$. By abuse of notation, we denote both the expression $v[0]v[1]\dots v[m](v[m+1]v[m+2]\dots v[n])^\omega$ and the value $DS(v[0]v[1]\dots v[m](v[m+1]v[m+2]\dots v[n])^\omega, d)$ by $v$.

We will construct a Büchi automaton for the comparison language $\mathcal{L}_\leq$ for relation $\leq$, threshold value $v \in \mathbb{Q}$ and an integer discount factor. This is sufficient to prove $\omega$-regularity for all relations since Büchi automata are closed.

From safety/co-safety of comparison languages, we argue it is sufficient to examine the discounted-sum of finite-length weight sequences to know if their infinite extensions will be in $\mathcal{L}_\leq$. For instance, if the discounted-sum of a finite-length weight-sequence $W$ is *very large*, $W$ could be a bad-prefix of $\mathcal{L}_\leq$. Similarly, if the discounted-sum of a finite-length weight-sequence $W$ is *very small* then for all of its infinite-length bounded extensions $Y$, $DS(W \cdot Y, d) \leq v$. Thus, a mathematical characterization of *very large* and *very small* would formalize a criterion for membership of sequences in $\mathcal{L}_\leq$ based on their finite-prefixes.

To this end, we use the concept of a *recoverable gap* (or gap value), which is a measure of distance of the discounted-sum of a finite-sequence from 0 [12]. The recoverable gap of a finite weight-sequences $W$ with discount factor $d$, denoted $\mathsf{gap}(W, d)$, is defined as follows: If $W = \varepsilon$ (the empty sequence), $\mathsf{gap}(\varepsilon, d) = 0$, and $\mathsf{gap}(W, d) = d^{|W|-1} \cdot DS(W, d)$ otherwise. Then, Lemma 3 formalizes *very large* and *very small* in Item 1 and Item 2, respectively, w.r.t. recoverable gaps. As for notation, given a sequence $A$, let $A[\dots i]$ denote its $i$-length prefix:

**Lemma 3.** *Let $\mu > 0$ be the integer upper bound, $d > 1$ be the discount factor. Let $v \in \mathbb{Q}$ be the threshold value s.t. $v = v[0]\dots v[m](v[m+1]\dots v[n])^\omega$. Let $W$ be a non-empty, bounded, finite-length weight-sequence.*

1. $\mathsf{gap}(W - v[\cdots |W|], d) > \frac{1}{d} \cdot DS(v[|W| \cdots], d) + \frac{\mu}{d-1}$. *iff for all infinite-length, bounded extensions $Y$, $DS(W \cdot Y, d) > v$*
2. $\mathsf{gap}(W - v[\cdots |W|], d) \leq \frac{1}{d} \cdot DS(v[|W| \cdots], d) - \frac{\mu}{d-1}$ *iff For all infinite-length, bounded extensions $Y$, $DS(W \cdot Y, d) \leq v$*

*Proof.* We present proof of one direction of Item 1. The others follow similarly. Let $W$ be s.t for every infinite-length, bounded $Y$, $DS(W \cdot Y, d) > v$ holds. Then $DS(W, d) + \frac{1}{d^{|W|}} \cdot DS(Y, d) \geq DS(v[\cdots |W|] \cdot v[|W| \cdots], d)$ implies $DS(W, d) - DS(v[\cdots |W|], d) > \frac{1}{d^{|W|}} \cdot (DS(v[|W| \cdots], d) - DS(Y, d))$ implies $\mathsf{gap}(W - v[\cdots |W|], d) > \frac{1}{d}(DS(v[|W| \cdots], d) + \frac{\mu \cdot d}{d-1})$. □

This segues into the state-space of the Büchi automaton. We define the state space so that state $s$ represents the gap value $s$. The idea is that all finite-length weight sequences with gap value $s$ will terminate in state $s$. To assign transition between these states, we observe that gap value is defined inductively as follows: $\mathsf{gap}(\varepsilon, d) = 0$ and $\mathsf{gap}(W \cdot w, d) = d \cdot \mathsf{gap}(W, d) + w$, where $w \in \{-\mu, \dots, \mu\}$. Thus there is a transition from state $s$ to state $t$ on $a \in \{-\mu, \dots, \mu\}$ if $t = d \cdot s + a$. Since $\mathsf{gap}(\varepsilon, d) = 0$, state 0 is assigned to be the initial state.

The issue with this construction is it has infinite states. To limit that, we use Lemma 3. Since Item 1 is a necessary and sufficient criteria for bad prefixes of safety language $\mathcal{L}_{\leq}$, all states with value larger than Item 1 are fused into one non-accepting sink. For the same reason, all states with gap value less than Item 1 are accepting states. Due to Item 2, all states with value less than Item 2 are fused into one accepting sink. Finally, since $d$ is an integer, gap values are integral. Thus, there are only finitely many states between Item 2 and Item 1.

**Theorem 6.** *Let $\mu > 0$ be an integer upper bound, $d > 1$ an integer discount factor, $\mathsf{R}$ an equality or inequality relation, and $v \in \mathbb{Q}$ the threshold value with an $n$-length representation given by $v = v[0]v[1]\dots v[m](v[m+1]v[m+2]\dots v[n])^\omega$.*

1. *The DS comparator automata for $\mu, d, \mathsf{R}, v$ is $\omega$-regular iff $d$ is an integer.*
2. *For integer discount factors, the DS comparator is a safety or co-safety automaton with $\mathcal{O}(\frac{\mu \cdot n}{d-1})$ states.*

*Proof.* To prove Item 1 we present the construction of an $\omega$-regular comparator automaton for integer upper bound $\mu > 0$, integer discount factor $d > 1$, inequality relation $\leq$, and threshold value $v \in \mathbb{Q}$ s.t. $v = v[0]v[1]\dots v[m](v[m+1]v[m+2]\dots v[n])^\omega$. , denoted by $\mathcal{A} = (S, s_I, \Sigma, \delta, \mathcal{F})$ where:

For $i \in \{0, \dots, n\}$, let $\mathsf{U}_i = \frac{1}{d} \cdot DS(v[i \cdots], d) + \frac{\mu}{d-1}$ (Lemma 3, Item 1)

For $i \in \{0, \dots, n\}$, let $\mathsf{L}_i = \frac{1}{d} \cdot DS(v[i \cdots], d) - \frac{\mu}{d-1}$ (Lemma 3, Item 2)

- $S = \bigcup_{i=0}^{n} S_i \cup \{\mathsf{bad}, \mathsf{veryGood}\}$ where $S_i = \{(s,i) | s \in \{\lfloor \mathsf{L}_i \rfloor + 1, \dots, \lfloor \mathsf{U}_i \rfloor\}\}$
- Initial state $s_I = (0,0)$, Accepting states $\mathcal{F} = S \setminus \{\mathsf{bad}\}$
- Alphabet $\Sigma = \{-\mu, -\mu+1, \dots, \mu-1, \mu\}$
- Transition function $\delta \subseteq S \times \Sigma \to S$ where $(s,a,t) \in \delta$ then:
  1. If $s \in \{\mathsf{bad}, \mathsf{veryGood}\}$, then $t = s$ for all $a \in \Sigma$
  2. If $s$ is of the form $(p,i)$, and $a \in \Sigma$
     (a) If $d \cdot p + a - v[i] > \lfloor \mathsf{U}_i \rfloor$, then $t = \mathsf{bad}$
     (b) If $d \cdot p + a - v[i] \leq \lfloor \mathsf{L}_i \rfloor$, then $t = \mathsf{veryGood}$
     (c) If $\lfloor \mathsf{L}_i \rfloor < d \cdot p + a - v[i] \leq \lfloor \mathsf{U}_i \rfloor$,
        i. If $i == n$, then $t = (d \cdot p + a - v[i], m+1)$
        ii. Else, $t = (d \cdot p + a - v[i], i+1)$

We skip proof of correctness as it follows from the above discussion. Observe, $\mathcal{A}$ is deterministic. It is a safety automaton as all non-accepting states are sinks.

To prove Item 2, observe that since the comparator for $\leq$ is a deterministic safety automaton, the comparator for $>$ is obtained by simply flipping the accepting and non-accepting states. This is a co-safety automaton of the same size. One can argue similarly for the remaining relations. □

### 4.2 Satisficing via safety and reachability games

This section describes our comparator-based linear-time algorithm for satisficing for integer discount factors.

As described earlier, given discount factor $d > 1$, a play is winning for satisficing with threshold value $v \in \mathbb{Q}$ and relation $\mathsf{R}$ if its cost sequence $A$ satisfies $DS(A, d)\ \mathsf{R}\ v$. We now know from Theorem 6, that the winning condition for plays can be expressed as a safety or co-safety automaton for any $v \in \mathbb{Q}$ as long as the discount factor is an integer. Therefore, a *synchronized product* of the quantitative game with the safety or co-safety comparator denoting the winning condition completes the reduction to a safety or reachability game, respectively.

**Theorem 7.** *Let $G = (V, v_{\mathsf{init}}, E, \gamma)$ be a quantitative game, $d > 1$ the integer discount factor, $\mathsf{R}$ the equality or inequality relation, and $v \in \mathbb{Q}$ the threshold value with an $n$-length representation. Let $\mu > 0$ be the maximum of absolute values of costs along transitions in $G$. Then,*

1. *The satisficing problem reduces to solving a safety game if* $\mathsf{R} \in \{\leq, \geq\}$
2. *The satisficing problem reduces to solving a reachability game if* $\mathsf{R} \in \{<, >\}$
3. *The satisficing problem is solved in $\mathcal{O}((|V| + |E|) \cdot \mu \cdot n)$ time.*

*Proof.* The first two points use a standard synchronized product argument on the following formal reduction [15]: Let $G = (V = V_0 \uplus V_1, v_{\mathsf{init}}, E, \gamma)$ be a quantitative game, $d > 1$ the integer discount factor, $\mathsf{R}$ the equality or inequality relation, and $v \in \mathbb{Q}$ the threshold value with an $n$-length representation. Let $\mu > 0$ be the maximum of absolute values of costs along transitions in $G$. Then, the first step is to construct the safety/co-safety comparator $\mathcal{A} = (S, s_I, \Sigma, \delta, \mathcal{F})$ for $\mu$, $d$, $\mathsf{R}$ and $v$. The next is to synchronize the product of $G$ and $\mathcal{A}$ over weights to construct the game $\mathsf{GA} = (W = W_0 \cup W_1, s_0 \times \mathsf{init}, \delta_W, \mathcal{F}_W)$, where

– $W = V \times S$. In particular, $W_0 = V_0 \times S$ and $W_1 = V_1 \times S$. Since $V_0$ and $V_1$ are disjoint, $W_0$ and $W_1$ are disjoint too.
– Let $s_0 \times \mathsf{init}$ be the initial state of $\mathsf{GA}$.
– Transition relation $\delta_W = W \times W$ is defined such that transition $((v, s), (v', s')) \in \delta_W$ synchronizes between transitions $(v, v') \in \delta$ and $(s, a, s') \in \delta_C$ if $a = \gamma((v, v'))$ is the cost of transition in $G$.
– $\mathcal{F}_W = V \times \mathcal{F}$. The game is a safety game if the comparator is a safety automaton and a reachability game if the comparator is a co-safety automaton.

We need the size of $\mathsf{GA}$ to analyze the worst-case complexity. Clearly, $\mathsf{GA}$ consists of $\mathcal{O}(|V| \cdot \mu \cdot n)$ states. To establish the number of transitions in $\mathsf{GA}$, observe that every state $(v, s)$ in $\mathsf{GA}$ has the same number of outgoing edges as state $v$ in $G$ because the comparator $\mathcal{A}$ is deterministic. Since $\mathsf{GA}$ has $\mathcal{O}(\mu \cdot n)$ copies of every state $v \in G$, there are a total of $\mathcal{O}(|E| \cdot \mu \cdot n)$ transitions in $\mathsf{GA}$. Since $\mathsf{GA}$ is either a safety or a reachability game, it is solved in linear-time to its size. Thus, the overall complexity is $\mathcal{O}((|V| + |E|) \cdot \mu \cdot n)$. □

With respect to the value $\mu$, the VI-based solutions are logarithmic in the worst case, while comparator-based solution is linear due to the size of the comparator. From a practical perspective, this may not be a limitation since weights along transitions can be scaled down. The parameter that cannot be altered is the size of the quantitative game. With respect to that, the comparator-based

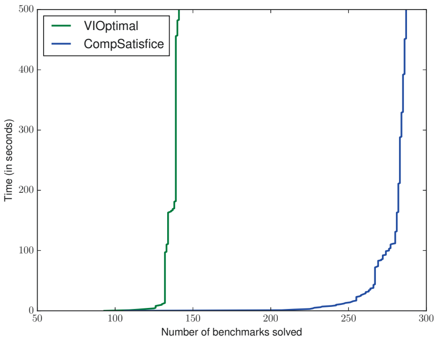

**Fig. 2.** Cactus plot. $\mu = 5, v = 3$. Total benchmarks = 291

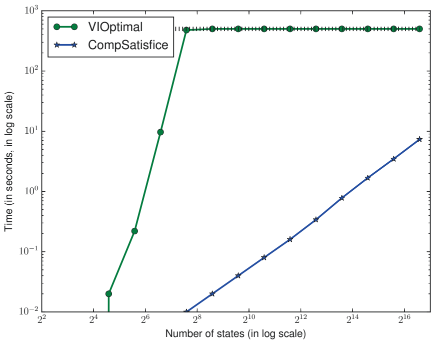

**Fig. 3.** Single counter scalable benchmark. $\mu = 5, v = 3$. Timeout = 500s.

solution displays clear superiority. Finally, the comparator-based solution is affected by $n$, length of the representation of the threshold value while the VI-based solution does not. It is natural to assume that the value of $n$ is small.

### 4.3 Implementation and Empirical Evaluation

The goal of the empirical analysis is to determine whether the practical performance of these algorithms resonate with our theoretical discoveries.

For an apples-to-apples comparison, we implement three algorithms: (a) VIOptimal: Optimization via value-iteration, (b)VISatisfice: Satisficing via value-iteration, and (c). CompSatisfice: Satisficing via comparators. All tools have been implemented in C++. To avoid floating-point errors in VIOptimal and VISatisfice, the tools invoke the open-source GMP (GNU Multi-Precision) [2]. Since all arithmetic operations in CompSatisfice are integral only, it does not use GMP.

To avoid completely randomized benchmarks, we create ∼290 benchmarks from $\mathsf{LTL_f}$ benchmark suite [29]. The state-of-the-art $\mathsf{LTL_f}$-to-automaton tool Lisa [8] is used to convert $\mathsf{LTL_f}$ to (non-quantitative) graph games. Weights are randomly assigned to transitions. The number of states in our benchmarks range from 3 to 50000+. Discount factor $d = 2$, threshold $v \in [0 - 10]$. Experiments were run on 8 CPU cores at 2.4GHz, 16GB RAM on a 64-bit Linux machine.

**Observations and Inferences** Overall, we see that VISatisfice is efficient and scalable, and exhibits steady and predictable performance.

CompSatisfice *outperforms* VIOptimal in both runtime and number of benchmarks solved, as shown in Fig 2. It is crucial to note that all benchmarks solved by VIOptimal had fewer than 200 states. In contrast, CompSatisfice solves much larger benchmarks with 3-50000+ number of states.

To test scalability, we compared both tools on a set of scalable benchmarks. For integer parameter $i > 0$, the $i$-th scalable benchmark has $3 \cdot 2^i$ states. Fig 3

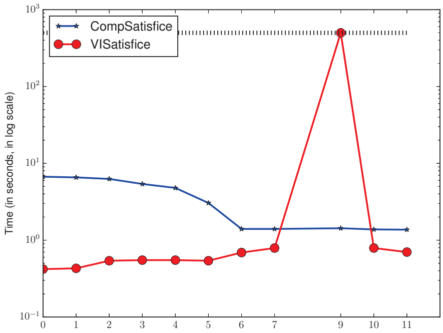

**Fig. 4.** Robustness. Fix benchmark, vary $v$. $\mu = 5$. Timeout = 500s.

plots number-of-states to runtime in log-log scale. Therefore, the slope of the straight line will indicate the degree of polynomial (in practice). It shows us that CompSatisfice exhibits linear behavior (slope $\sim$1), whereas VIOptimal is much more expensive (slope >> 1) even in practice.

CompSatisfice *is more robust than* VISatisfice*.* We compare CompSatisfice and VISatisfice as the threshold value changes. This experiment is chosen due to Theorem 4 which proves that VISatisfice is non-robust. As shown in Fig 4, the variance in performance of VISatisfice is very high. The appearance of peak close to the optimal value is an empirical demonstration of Theorem 4. On that other hand, CompSatisfice stays steady in performance owning to its low complexity.

## 5 Adding Temporally Extended Goals

Having witnessed algorithmic improvements of comparator-based satisficing over VI-based algorithms, we now shift focus to the question of applicability. While this section examines this with respect to the ability to extend to temporal goals, this discussion highlights a core strength of comparator-based reasoning in satisficing and shows its promise in a broader variety of problems.

The problem of extending optimal/satisficing solutions with a temporal goal is to determine whether there exists an optimal/satisficing solution that also satisfies a given temporal goal. Formally, given a quantitative game $G$, a labeling function $\mathcal{L} : V \to 2^{AP}$ which assigns states $V$ of $G$ to atomic propositions from the set $AP$, and a temporal goal $\varphi$ over $AP$, we say a *play* $\rho = v_0 v_1 \dots$ *satisfies* $\varphi$ if its proposition sequence given by $\mathcal{L}(v_0)\mathcal{L}(v_1)\dots$ satisfies the formula $\varphi$. Then to solve *optimization/satisficing with a temporal goal* is to determine if there exists a solutions that is optimal/satisficing and also satisfies the temporal goal along resulting plays. Prior work has proven that the optimization problem cannot be extended to temporal goals [13] unless the temporal goals are very simple safety properties [10,31]. In contrast, our comparator-based solution for satisficing can naturally be extended to temporal goals, in fact to all $\omega$-regular properties, owing to its automata-based underpinnings, as shown below:

**Theorem 8.** *Let $G$ a quantitative game with state set $V$, $\mathcal{L} : V \to 2^{AP}$ be a labeling function over set of atomic propositions $AP$, and $\varphi$ be a temporal goal over $AP$ and $\mathcal{A}_\varphi$ be its equivalent deterministic parity automaton. Let $d > 1$ be an integer discount factor, $\mu$ be the maximum of the absolute values of costs along transitions, and $v \in Q$ be the threshold value with an $n$-length representation. Then, solving satisficing with temporal goals reduces to solving a parity game of size linear in $|V|$, $\mu$, $n$ and $|\mathcal{A}_\varphi|$.*

*Proof.* The reduction involves two steps of synchronized products. The first reduces the satisficing problem to a safety/reachability game while preserving the labelling function. The second synchronization product is between the safety/reachability game with the DPA $\mathcal{A}_\varphi$. These will synchronize on the atomic propositions in the labeling function and DPA transitions, respectively. Therefore, resulting parity game will be linear in $|V|$, $\mu$ and $n$, and $|\mathcal{A}_\varphi|$. □

Broadly speaking, our ability to solve satisficing via automata-based methods is a key feature as it propels a seamless integration of quantitative properties (threshold bounds) with qualitative properties, as both are grounded in automata-based methods. VI-based solutions are inhibited to do so since numerical methods are known to not combine well with automata-based methods which are so prominent with qualitative reasoning [5,20]. This key feature could be exploited in several other problems to show further benefits of comparator-based satisficing over optimization and VI-based methods.

## 6 Concluding remarks

This work introduces the satisficing problem for quantitative games with the discounted-sum cost model. When the discount factor is an integer, we present a comparator-based solution for satisficing, which exhibits algorithmic improvements – better worst-case complexity and efficient, scalable, and robust performance – as well as broader applicability over traditional solutions based on numerical approaches for satisficing and optimization. Other technical contributions include the presentation of the missing proof of value-iteration for optimization and the extension of comparator automata to enable direct comparison to arbitrary threshold values as opposed to zero threshold value only.

An undercurrent of our comparator-based approach for satisficing is that it offers an automata-based replacement to traditional numerical methods. By doing so, it paves a way to combine quantitative and qualitative reasoning without compromising on theoretical guarantees or even performance. This motivates tackling more challenging problems in this area, such as more complex environments, variability in information availability, and their combinations.

**Acknowledgements.** We thank anonymous reviewers for valuable inputs. This work is supported in part by NSF grant 2030859 to the CRA for the CIFellows Project, NSF grants IIS-1527668, CCF-1704883, IIS-1830549, the ERC CoG 863818 (ForM-SMArt), and an award from the Maryland Procurement Office.
